# An efficient protocol for functional studies of apple transcription factors using a glucocorticoid receptor fusion system

**DOI:** 10.1002/aps3.11396

**Published:** 2020-10-30

**Authors:** Joan Estevan, Sara Gómez‐Jiménez, Vítor da Silveira Falavigna, Alicia Camuel, Lisa Planel, Evelyne Costes, Fernando Andrés

**Affiliations:** ^1^ AGAP University of Montpellier CIRAD INRAE Institut Agro Montpellier France; ^2^ Present address: Max Planck Institute for Plant Breeding Research Carl‐von‐Linne‐Weg 10 50829 Cologne Germany

**Keywords:** apple, GR system, MdFLC, target gene identification, transcription factor

## Abstract

**Premise:**

We report a protocol for studying the function of apple (*Malus* ×*domestica*) transcription factors based on the glucocorticoid receptor (GR) system, which allows the dexamethasone (DEX)‐mediated activation of plant transcription factors to monitor the expression levels of their potential target genes.

**Methods and Results:**

Apple leaves are transformed with a vector that allows the expression of the studied transcription factor (i.e., FLOWERING LOCUS C [MdFLC]) fused to GR. Calli derived from the transformed leaves are treated with DEX and cycloheximide, a protein synthesis inhibitor. Compared with other methods, combining the GR system with cycloheximide treatments enables the differentiation between direct and indirect transcription factor target genes. Finally, the expression levels of putative *MdFLC* target genes are quantified using quantitative reverse transcription PCR.

**Conclusions:**

We demonstrate the efficiency of our GR system to study the function of apple transcription factors. This method is accessible to any laboratory familiar with basic molecular cloning procedures and the apple leaf–mediated agro‐transformation technique.

Apple (*Malus* ×*domestica* Borkh.) belongs to the Malaceae subfamily of the Rosaceae, and is one of the most important fruit crops in terms of worldwide production and consumption (Loiseau et al., 2020). For this reason, several genetic resources and molecular techniques have been developed over the past three decades to facilitate the study of fundamental biological questions related to apple agronomical and commercial traits. Notably, the apple genome sequence was completed in 2010, with other high‐quality genomes being released since then (Velasco et al., 2010; Daccord et al., 2017; Zhang et al., 2019; Broggini et al., 2020). Furthermore, efficient apple genetic transformation protocols have been developed over the past 30 years (James et al., 1989, 1993; De Bondt et al., 1994; Norelli et al., 1996; Puite and Schaart, 1996; Malnoy et al., 2010; Charrier et al., 2019); most of these protocols are based on *Agrobacterium tumefaciens*–mediated transformation and, therefore, are commonly used in several laboratories (James et al., 1989).

Inducible gene expression systems using chemical inducers are tremendously useful for performing basic research in functional genomics. These systems allow the temporal and spatial control of gene expression patterns to elucidate gene functions. This is especially interesting in the study of the function of transcription factors (TFs), enabling the identification of their target genes and the gene regulatory networks in which they are involved. A popular system to study TF functions in plants is the glucocorticoid receptor (GR) system. Upon dexamethasone (DEX) treatment, a TF fused to the GR is translocated to the nucleus from the cytoplasm, where it binds to its target genes to (potentially) regulate their transcription (i.e., their repression or activation) (Aoyama and Chua, 1997). The GR system was shown to be efficient for characterizing TF functions not only in entire transgenic plants, but also in excised tissues and single cells (Bargmann et al., 2013). This is particularly useful for plants with a low transformation efficiency, long regeneration times, and a long lifecycle. This is the case for most fruit trees, which require at least several months to be genetically transformed and a few years to reach the adult phase (Prieto, 2011).

The apple *FLOWERING LOCUS C* gene (*MdFLC*) encodes a MADS‐box TF believed to participate in the control of the dormancy cycle (Porto et al., 2015). In order to shed light on the role of the MdFLC TF, we identified its genome‐wide binding sites using DNA affinity purification sequencing (DAP‐seq) (V. S. Falavigna, E. Severing, X. Lai, J. Estevan, V. Hugouvieux, I. Farrera, F. Parcy, et al., unpublished data; O’Malley et al., 2016), and thus, its putative transcriptional targets. In this paper, we report a new protocol based on the GR system that was developed to study the effect of TFs on the transcriptional regulation of their target genes. As a proof of concept, we investigated the effect of the TF MdFLC on the expression of the putative target genes identified in our DAP‐seq experiment. To this end, we transformed apple calli with a vector expressing MdFLC TF fused to the GR protein and used quantitative reverse transcription PCR (RT‐qPCR) to monitor the effect of the target gene induction by a DEX treatment. These experiments were performed in the presence of the protein synthesis inhibitor cycloheximide, favoring the detection of direct target genes. Our results indicate that the TF‐GR protocol presented here is an efficient and reliable method to evaluate the MdFLC TF target genes, and is therefore a valuable tool in gene functional studies.

## METHODS AND RESULTS

### Vector construction and transformation in *Agrobacterium tumefaciens*

**FIGURE 1:**
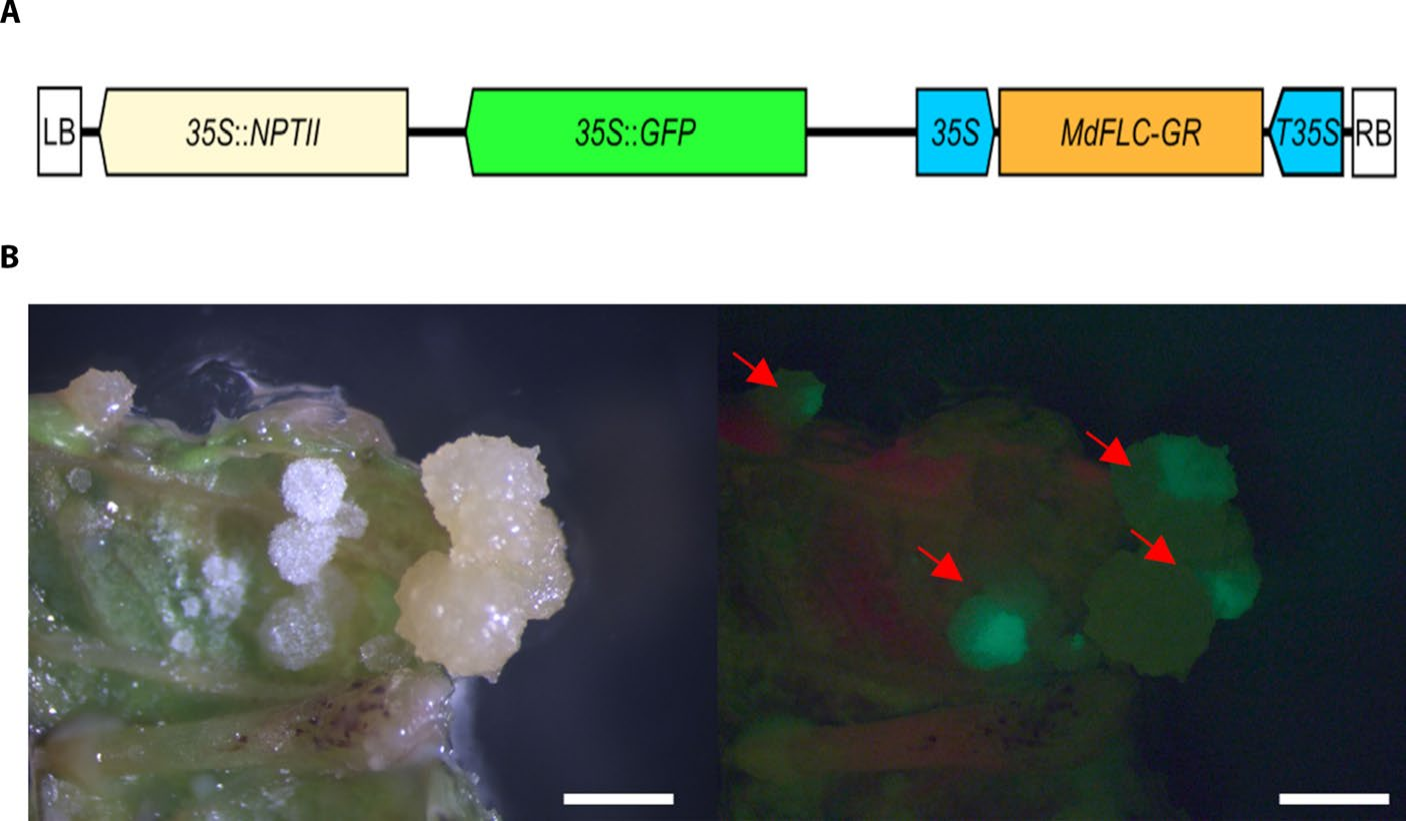
Construction of the pCamway35‐*MdFLC*‐*GR* expression vector and identification of successfully transformed apple calli. (A) Schematic representation of the pCamway35S vector harboring the *35S*::*MdFLC*‐*GR* cassette (pCamway35‐*MdFLC*‐*GR*). pCamway35S is a pCambia2300‐GFP backbone‐based vector (Leclercq et al., 2015). LB, left T‐DNA border; 35S, CaMV *35S* promoter; *NPTII*, *neomycin phosphotransferase*; *GFP*, *GREEN FLUORESCENT PROTEIN*; T35S, T35S terminator; RB, right T‐DNA border. (B) Binocular observation of calli from leaves four weeks after their transformation with the pCamway35‐*MdFLC*‐*GR* vector, both under white light (left) and GFP fluorescence filter (right). In the image on the right, GFP‐positive calli are indicated with a red arrow. Scale bars: 1 mm.

The complete coding sequence of *MdFLC* (MD09G1009100) cloned into the pENTR/D‐TOPO vector was provided by Dr. Luís F. Revers (Embrapa Uva e Vinho, Bento Gonçalves, Brazil). Gene‐specific primers (Table 1) were used to amplify the coding sequence of *MdFLC* without the stop codon using KOD Hot Start DNA Polymerase (Merck, Darmstadt, Germany). The PCR protocol consisted of one step at 95°C for 2 min, followed by 40 cycles at 95°C for 20 s, 60°C for 10 s, and 70°C for 10 s. The PCR product was visualized in a 1% agarose gel and purified using a Zymoclean Gel DNA Recovery Kit (Zymo Research, Irvine, California, USA). The purified PCR product was cloned into the pDONR207 vector (Karimi et al., 2007) using Gateway BP Clonas II Enzyme Mix (Thermo Fisher Scientific, Waltham, Massachusetts, USA), according to the manufacturer’s instructions. This construct was recombined into the pBEACON‐GR vector (Bargmann et al., 2013), provided by Dr. Gloria Coruzzi (New York University, New York, New York, USA), using Gateway LR Clonas II Enzyme Mix (Thermo Fisher Scientific). The *MdFLC*‐*GR* sequence was amplified and purified as described above using the primers FLC‐Fw‐BP and GR‐Rv2‐BP (Table 1). The purified *MdFLC*‐*GR* PCR product was cloned into the pDONR207 vector as described above. The resulting vector was recombined into the binary vector pCamway35S (Leclercq et al., 2015), which carries the in planta selection marker *35S*::*GFP* (*GREEN FLUORESCENT PROTEIN*) and an additional constitutive‐expression promoter CaMV *35S* to drive the expression of *MdFLC‐GR*. The structure of the final vector (pCamway35‐*MdFLC*‐*GR*) is shown in Fig. 1A. The vectors resulting from each different cloning step were confirmed by sequencing. The final expression vector harboring the *35S*::*MdFLC*‐*GR* cassette was used in the transformation of the *A. tumefaciens* strain EHA105 (Hood et al., 1993).

**TABLE 1:** List of primers used in this study.

Purpose	Primer name	Accession code^a^	Primer sequence (5′–3′)
Molecular cloning	FLC‐Fw‐BP	MD09G1009100	GGGGACAAGTTTGTACAAAAAAGCAGGCTCTAT GGGGCGAGGGAAGGTGCAG
	FLC‐Rv‐BP‐nostop2	MD09G1009100	GGGGACCACTTTGTACAAGAAAGCTGGGTTAAAC AACTGTAGTATGGTGGCCG
	GR‐Rv2‐BP	pBEACON‐GR	GGGGACCACTTTGTACAAGAAAGCTGGGTCTAGC ATGGCCGTTTTTGAT
Expression studies	WD40‐F	MD08G1215900	GGATTTACTGTGTTGGTGAAG
	WD40‐R	MD08G1215900	TGCCAATTACCTCCTTTTCGTG
	MdFLC‐like‐F	MD09G1009100	AACAGATGAAAGAAGAGAAGGTTCG
	MdFLC‐like‐R	MD09G1009100	TATTAGCGGCGGAAGTGCTC
	MDH‐F	MD16G1219000	CGTGATTGGGTACTTGGAAC
	MDH‐R	MD16G1219000	TGGCAAGTGACTGGGAATGA
	HAI3‐F	MD01G1220800	TGGGAGAGTCATCAACTGGA
	HAI3‐R	MD01G1220800	ACCGTCACCTCTGGTCTTG
	FUL‐F	MD09G1074000	GGAGGAGTGGATTGCTCAAA
	FUL‐R	MD09G1074000	CCCTACGGTGGAGAAGACAA
	ABA2‐F	MD07G1033200	CGGGTTCGCTTCCAAA
	ABA2‐R	MD07G1033200	GGTTGTCCTGCACATCAACTAG
	GASA4‐F	MD17G1041500	CATGGGCATGGAGGTCAT
	GASA4‐R	MD17G1041500	GGCACACACAGGCACTTT

^a^ Nomenclature of accession codes from the apple genome GDDH13 version 1.1 (Daccord et al., 2017).

### Production of transformed calli from leaf explants

The apple cultivar ‘Gala’ was used in this study. In vitro cuttings of apple were subcultured at one‐month intervals on a micropropagation medium, a modified Lepoivre medium (Leblay et al., 1991) supplemented with 0.5 mg/L 6‐benzylaminopurine and 0.1 mg/L indole‐3‐butyric acid, at 22°C under a 16‐h light (80 µmol m^−2^ s^−1^)/8‐h dark photoperiod. Previously described apple leaf transformation protocols (James et al., 1993) were modified and used to produce transformed calli that could be easily stored and multiplied for TF functional studies. Transformed *A*. *tumefaciens* was grown for 48 h on a Luria–Bertani solid medium containing 50 µg/mL kanamycin. The bacteria inoculum at OD_600_ = 1 was resuspended in an induction medium containing 150 µM acetosyringone (James et al., 1993) and incubated at room temperature with gentle agitation for 3–4 h. The day before the transformation, the youngest leaves of four‐week‐old micropropagated shoots were harvested (from axillary shoot cultures) and incubated in the dark on a regeneration medium, which was a modified Lepoivre medium (see above) supplemented with 5 mg/L thidiazuron and 0.2 mg/L α‐naphtalene acetic acid. The leaves were wounded perpendicularly to the midrib using a scalpel (two or three internal sections) and vacuum‐infiltrated for 1 min with the inoculum at −0.09 mPa. The inoculated leaves were placed abaxial surface up on a co‐cultivation medium (regeneration medium with the addition of 100 µM acetosyringone), and the plates were incubated in the dark at 22°C for 48 h. After co‐cultivation, the leaves were transferred, adaxial surface up, onto a selection/callogenesis medium, a modified Lepoivre medium (see above) supplemented with 0.5 mg/L of kinetin, 0.5 mg/L 2,4*‐*dichlorophenoxyacetic acid, 50 mg/L kanamycin, 300 mg/L cefotaxime, and 150 mg/L ticarcillin disodium, before being placed in darkness at 22°C. One month after transformation, the putatively transformed calli that proliferate in the presence of antibiotics were observed under a fluorescence stereo zoom microscope (Leica MZFLIII; Leica Camera AG, Wetzlar, Germany) (Fig. 1B). The GFP‐expressing calli were further selected and subcultured into the selection medium at three‐week intervals in the dark at 22°C. Six months after the leaf transformation, about 30 transformed calli (approximatively 5 mm in diameter) were obtained.

### Chemical induction of expression

Successfully transformed calli were subjected to different treatments, while non‐transformed calli of a similar age were used as wild‐type controls. Each sample comprised a pool of 10 calli. One wild‐type and one transformed sample (T0) of calli were frozen in liquid nitrogen at the beginning of the assay (no treatment) and stored at −80°C. The other calli samples were treated with DEX to induce the nuclear accumulation of MdFLC TF, as described elsewhere (Schena and Yamamoto, 1988; Bargmann et al., 2013). A list of required materials and reagents is provided in Appendix 1. Transformed calli were pretreated with 40 μM cycloheximide for 30 min and then rinsed with distilled water. Subsequently, 10 μM DEX was added to the treated samples (+DEX) and incubated at room temperature for 1 h before being rinsed with distilled water and stored at room temperature for 3 (T4) or 7 (T8) h. Negative controls (−DEX) were treated with solvent alone (i.e., the 95% ethanol used to dissolve DEX). Calli treated with DEX or the solvent were collected and frozen at −80°C.

### Expression studies

Total RNA was isolated using a commercial kit (Spectrum Plant Total RNA kit; Sigma‐Aldrich, St. Louis, Missouri, USA) following the manufacturer’s instructions. The integrity of the total RNA was analyzed using a 4200 TapeStation instrument (Agilent Technologies, Santa Clara, California, USA), while the RNA quantification was performed with a NanoQuant instrument (Infinite 200 NanoQuant; Tecan, Männedorf, Switzerland). The RNA was subjected to a DNase treatment using a Turbo DNA‐free kit (Thermo Fisher Scientific), and cDNA was synthesized from the RNA template using a Superscript III Reverse Transcriptase kit (Thermo Fisher Scientific), following the manufacturer’s instructions.

For the RT‐qPCR reactions, 2 μL of the cDNA samples (diluted 1 : 10) was used as a template in a 6‐μL final reaction volume containing 3 μL of 2× LightCycler 480 SYBR Green I Master mix (Roche, Basel, Switzerland) and 3 μM of each primer. The real‐time PCR reactions were run on a LightCycler 480 (Roche) with an initial denaturation step of 5 min at 95°C followed by 40 cycles of 20 s at 95°C, 20 s at 60°C, and 20 s at 72°C. The PCR products were analyzed in a melting curve analysis to verify the presence of a gene‐specific PCR product. The melting curve analysis was performed immediately after the PCR amplification using a single step at 95°C for 1 min, 40°C for 1 min, and an annealing procedure starting at 65°C and increasing to 95°C at increments of 0.02°C/s. Each reaction included negative and positive controls and each cDNA sample was analyzed in three technical replicates. In a pilot experiment, two housekeeping genes—*WD‐40 repeat family protein* (*WD40*) and *malate dehydrogenase* (*MDH*)—were used to normalize the amount of plant RNA in each sample (Perini et al., 2014). The two housekeeping genes gave a very similar result, and thus only one of them, *WD40*, was used in further experiments (Appendix S1). The putative MdFLC TF target genes analyzed here were previously identified using DAP‐seq (Falavigna et al., unpublished data). The transcript levels were calculated with LightCycler 480 software version 1.5.0.39 (Roche), and the efficiency of the primers was determined using LinRegPCR (Ruijter et al., 2009). The ΔΔCt method (Livak and Schmittgen, 2001) was used to analyze the data. The list of primers used to quantify the expression of the genes of interest is provided in Table 1. A Student’s *t*‐test was performed to estimate the significance of the difference between the +DEX and –DEX treatments at each timepoint.

**FIGURE 2:**
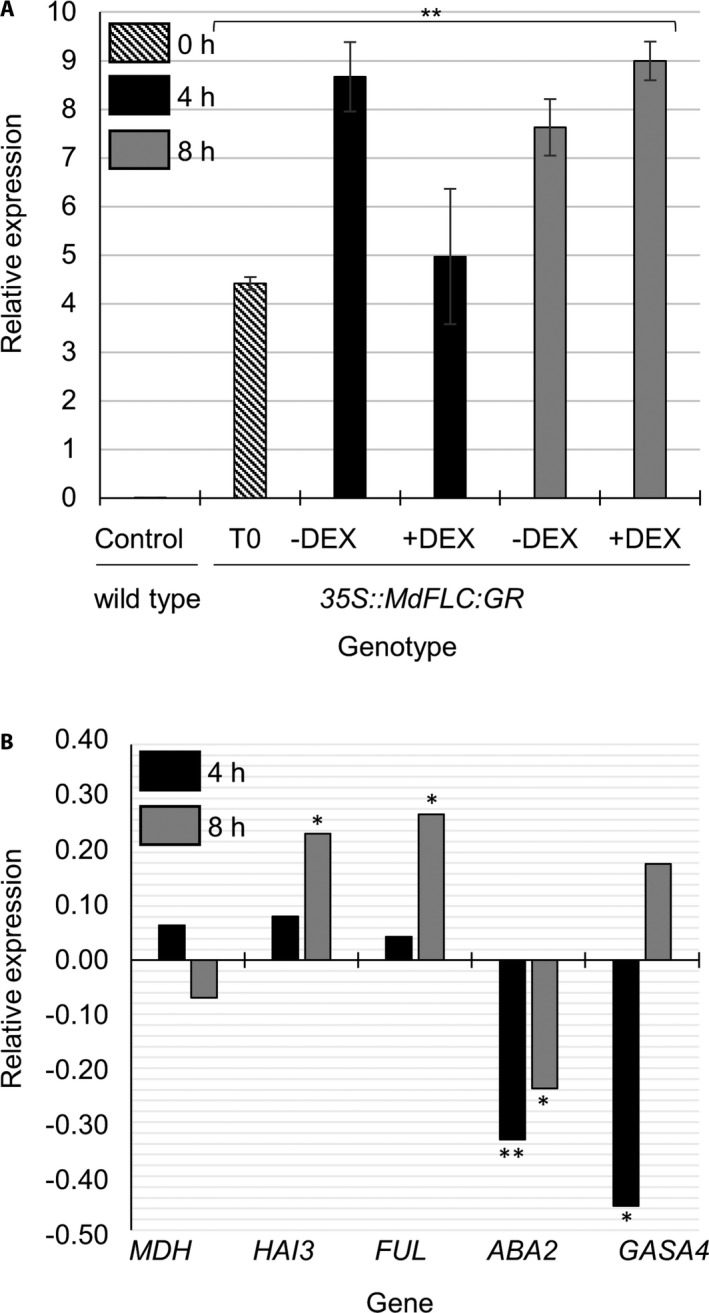
Gene expression levels in transformed apple calli upon treatment with dexamethasone (DEX). Gene expression levels, normalized with *WD40*, were quantified using RT‐qPCR in transformed calli treated with DEX (+DEX) or a mock solvent (−DEX) and collected after 0, 4, and 8 h for (A) and 4 and 8 h for (B). (A) Comparison of *MdFLC* gene expression in non‐transformed calli (control) and in calli transformed with *35S::MdFLC:GR*. Expression values are the mean from three technical replicates, and the error bars indicate the standard error. (B) The expression of MdFLC TF target genes in calli transformed with *35S::MdFLC::GR*. The results are shown as the log_2_ values of the ratio of the mean transcript levels for DEX vs. −DEX from three technical replicates. *MDH*, *malate dehydrogenase*; *HAI3*, *HIGHLY ABA‐INDUCED PP2C GENE 3*; *FUL*, *FRUITFULL*; *ABA2*, *ABA DEFICIENT 2*; *GASA4*, *GAST1 PROTEIN HOMOLOG 4*. For (A) and (B), statistical differences were calculated using Student’s *t*‐test (*P ≤ 0.01; **P ≤ 0.001).

The level of *MdFLC* mRNA expression in the calli samples was quantified to confirm the efficiency of its overexpression driven by the *35S* promoter. As shown in Fig. 2A, the expression levels of *MdFLC* were significantly higher in all transgenic calli compared with the control (non‐transformed) calli. The transcript levels of four potential target genes of MdFLC—*HIGHLY ABA‐INDUCED PP2C GENE 3* (*HAI3*), *FRUITFULL* (*FUL*), *ABA DEFICIENT 2* (*ABA2*), and *GAST1 PROTEIN HOMOLOG 4* (*GASA4*)—were studied using RT‐qPCR over two timepoints (4 h [T4] and 8 h [T8]) in the mock (−DEX) or DEX‐treated transformed calli (Fig. 2B, Appendix S1). The reference gene *MDH* (Perini et al., 2014) was used as a negative control (it is not bound by MdFLC TF). Notably, the expression levels of the four tested putative MdFLC TF target genes were altered by the DEX treatments in at least one of the assayed timepoints. The expression levels of *HAI3* and *FUL* were significantly higher in the +DEX samples compared with the −DEX samples at T8. On the other hand, the levels of *ABA2* expression at the two timepoints (T4 and T8) and *GASA4* at T4 were significantly downregulated in the +DEX samples compared with the −DEX treatment. The expression levels of the negative control gene *MDH* did not show a statistical difference at any of the assayed timepoints.

## CONCLUSIONS

Here, we report a protocol for functional genomics studies that facilitates the quantification of the expression of TF target genes using a GR system. This protocol has been demonstrated to be efficient and sensitive for the detection of changes in the expression of MdFLC TF target genes.

The putative MdFLC TF target genes interrogated in this study were previously identified using DAP‐seq (Falavigna et al., unpublished); however, in addition to the analysis of known TF target genes, this system can be applied to discover novel TF target genes in combination with RNA‐seq or ChIP‐seq techniques. Our system allows the rapid and timely TF activation by DEX, and thus enables the monitoring of the dynamic transcriptional changes of the target genes using time‐course experiments. This feature is instrumental for selecting the optimal conditions for transcriptomic experiments; for example, we observed that the expression levels of some MdFLC target genes were only significantly affected at one particular timepoint after DEX treatment (Fig. 2B). By contrast, other methods used in the study of apple TF function using ectopic expression protocols do not allow the fine‐tuning of TF activity and therefore might not be efficient in detecting genome‐wide TF target genes. Additionally, by making use of the translational inhibitor cycloheximide, it is possible to distinguish between direct and indirect TF target genes, which is not likely by relying solely on constitutive expression systems.

In conclusion, we have developed a protocol for functional studies of apple that can be easily performed in any laboratory familiar with basic molecular cloning procedures and the apple leaf–mediated agro‐transformation technique. Coupled with genome‐wide approaches, this protocol can be instrumental for unraveling the molecular function of apple TFs and for deciphering the gene regulatory networks in which they are involved.

## AUTHOR CONTRIBUTIONS

J.E., S.G‐J., and A.C. performed the genetic transformation experiments. V.S.F. performed the molecular cloning and vector construction. J.E. and L.P. performed the gene expression studies and analyzed the results. J.E. and F.A. conceived the protocol and designed the experiments. J.E., V.S.F., E.C., and F.A. wrote the manuscript. All the authors approved the final version.

## Supporting information


**APPENDIX S1.** Pilot experiment and gene expression levels in transformed apple calli upon treatment with dexamethasone (DEX).

## Data Availability

The authors confirm that all data underlying the findings are fully available without restriction. All data are included within the manuscript and Appendix S1.

## APPENDIX 1 Materials and reagents required to perform the chemical induction of MdFLC transcription factor nuclear accumulation.

### Chemicals/solutions

- 95% ethanol
- Dimethylsulfoxide (DMSO)
- Liquid nitrogen
- Distilled water
- Dexamethasone (DEX) (catalog no.: D4902; Sigma‐Aldrich, St. Louis, Missouri, USA): 1 mM DEX stock was dissolved in 95% ethanol and stored at −20°C
- Cycloheximide (catalog no.: C4859; Sigma‐Aldrich, St. Louis, Missouri, USA): a 50 mM cycloheximide stock was dissolved in DMSO and stored at −20°C

### Equipment

- Tweezers
- 10 µL and 100 µL micropipettes and tips
- 1.5 mL microcentrifuge tubes (catalog no.: 87003‐294; VWR International, Radnor, Pennsylvania, USA)
- −80°C freezer
- Fume hood
