# Supplementary material for: An efficient protocol for functional studies of apple transcription factors using a glucocorticoid receptor fusion system
Source: Appl Plant Sci. 2020 Oct 30;8(10):e11396. doi: 10.1002/aps3.11396 (PMC7598887; doi:10.1002/aps3.11396)

Estevan et al.—Applications in Plant Sciences 2020 8(10)—Data Supplement S1. Page 1 of 1.
DOI 10.1002/aps3.11396

APPENDIX S1. Pilot experiment and gene expression levels in transformed apple calli upon treatment with dexamethasone (DEX). Gene expression levels were quantified using RT-qPCR in transfomed apple calli treated with DEX (+DEX) or mock (–DEX) and collected after 4 and 8 h. Gene expression values are the mean from three technical replicates, and error bars indicate the standard error. Statistical differences were calculated using Student's *t*-test (*$P \leq 0.01$, **$P \leq 0.001$); asterisks represent differences between the two conditions (+DEX and –DEX) at the same collection time (4 and 8 h). (A) Comparison of *HAI* and *MdFLC* gene expression after normalization with *WD40* or *MDH*. (B) MdFLC transcription factor targets gene expression in *35S::MdFLC::GR* normalized with *WD40*.

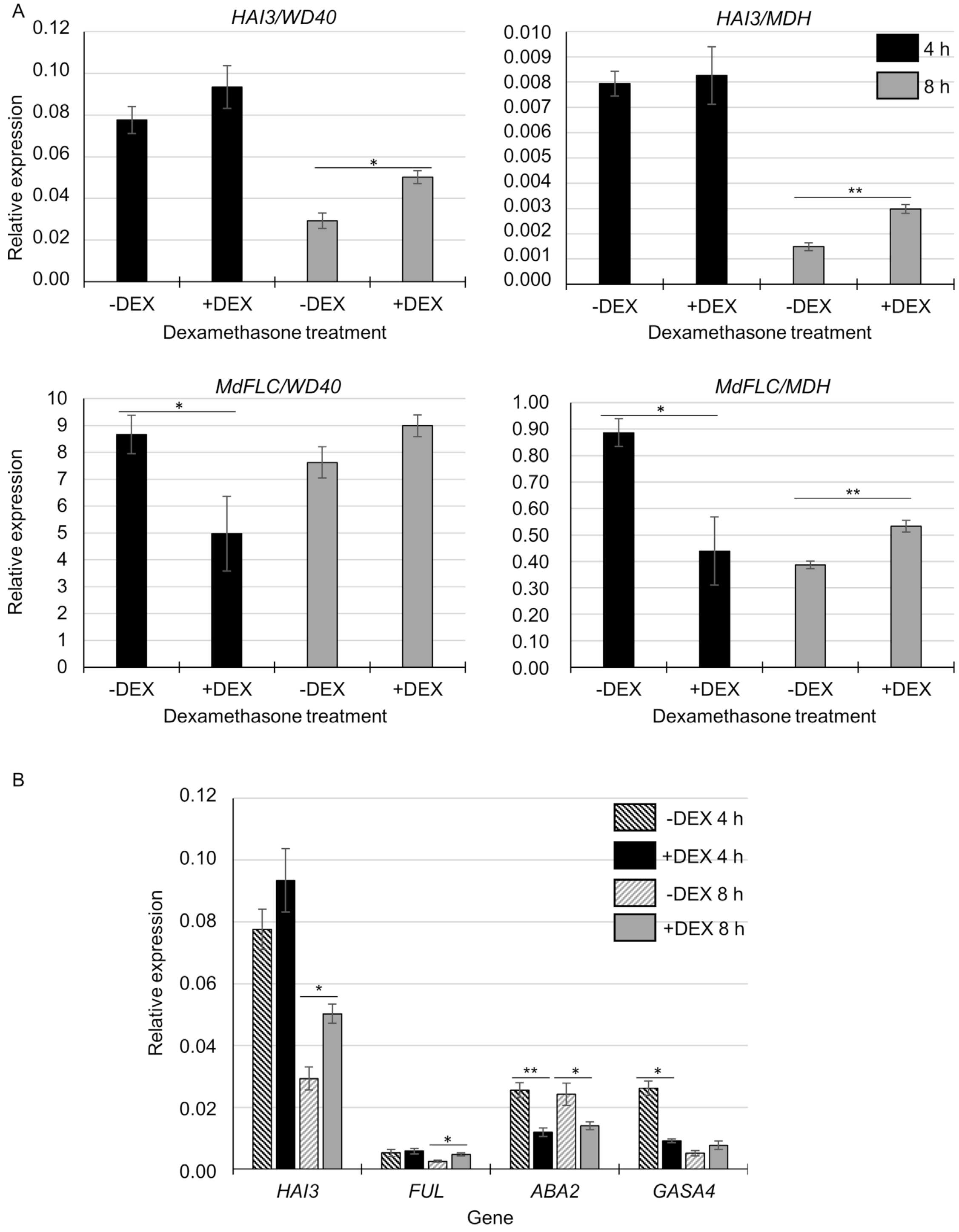

Supplement: Supplementary file 1 — APPENDIX S1. Pilot experiment and gene expression levels in transformed apple calli upon treatment with dexamethasone (DEX). [file APS3-8-e11396-s001.pdf]
